# COVID-19 in the Clinic: Aerosol Containment Mask for Endoscopic Otolaryngologic Clinic Procedures

**DOI:** 10.1177/01945998211024944

**Published:** 2021-06-22

**Authors:** Elisabeth H. Ference, Wihan Kim, John S. Oghalai, Jee-Hong Kim, Brian E. Applegate

**Affiliations:** 1 Caruso Department of Otolaryngology–Head and Neck Surgery, Keck School of Medicine of University of Southern California, Los Angeles, California, USA

**Keywords:** negative pressure mask, endoscopy, laryngoscopy, nasal endoscopy, aerosol production, COVID-19

## Abstract

**Objective:**

To create an aerosol containment mask (ACM) that contains aerosols during common otolaryngologic endoscopic procedures while protecting patients from environmental aerosols.

**Study Design:**

Bench testing.

**Setting:**

Mannequin testing.

**Methods:**

The mask was designed in SolidWorks and 3-dimensional printed. Mannequins were fitted with a nebulizer to generate aerosols. Commercial particle counters were used to measure mask performance.

**Results:**

The ACM has 2 ports on either side for instruments and endoscopes, a port for a filter, and a port that can evacuate aerosols contained within the mask via a standard suction pump. The mask contained aerosols on a mannequin with and without facial hair when the suction was set to 18.5 L/min. Other types of masks demonstrated substantial aerosol leakage under similar conditions. In a subsequent experiment, the ACM contained aerosols generated by a nebulizer up to the saturation of the particle detector without measurable leakage with or without suction.

**Conclusion:**

The ACM will accommodate rigid and flexible endoscopes plus instruments and prevent leakage of patient-generated aerosols, thus avoiding contamination of the room and protecting health care workers from airborne contagions.

**Level of evidence:**

2.

Due to the spread of SARS-CoV-2, the virus responsible for COVID-19, clinicians and hospitals face difficult decisions regarding how to provide care for patients in clinics during procedures that may lead to the generation of aerosols. Airborne SARS-CoV-2 has been found in hospital rooms and ventilation systems where patients with COVID-19 have been treated.^1-5^ Aerosolized particles <5 μm may remain viable in the air for at least 3 hours.^6^ While one study found that laryngoscopy alone may not generate aerosols greater than that produced by breathing, laryngoscopy and nasal endoscopy are associated with increased risk of coughing and sneezing, which are aerosol-generating events.^7,8^ Currently in many practices, patients wear a surgical mask over their mouths during nasal endoscopy, although a regular surgical mask is insufficient to protect at close range against all particle transmission generated by simulated aerosol generation.^8^

In principle, virus aerosolized during clinic procedures could infect not only the surgeon performing the procedure but others who enter the room. The Centers for Disease Control and Prevention recommends that procedure rooms without negative pressure remain vacant following any aerosol-generating procedure before undergoing deep cleaning. This period is typically deemed to be 6 times the room air turnover time.^9,10^ These cleaning and time requirements, compounded by limited testing capacity with variable time to results, can severely diminish an outpatient clinic’s capacity to treat patients.

Prior authors have suggested negative pressure microenvironments,^11^ modification of Ambu,^12^ nasotracheal intubation^13^ face masks with negative pressure, or a modified N95 mask^8^ to decrease aerosol dispersion during diagnostic nasal endoscopy and laryngoscopy. We present a 3-dimensional (3D) printed negative pressure respiratory aerosol containment mask (ACM) that provides N95-level protection to the patient. The negative pressure is generated through a standard suction commonly found in otolaryngology clinics. We measured aerosol generation in the ACM and compared it with previously described masks with and without instrumentation.

## Materials and Methods

The study was approved by the University of Southern California Institutional Review Board (HS-20-00482).

### Mask Design and Development

We created multiple design iterations by using SolidWorks (Dassault Systemes) and printing on a 3D printer (Ultimaker) with tough PLA (polylactic acid; Ultimaker). We tested initial prototypes on endoscopic surgery model heads to gauge access to the nasal cavity and ability to contain aerosols. The design was modified as issues were identified. The main considerations during the design phase were to appropriately position the blind grommet, find a gel cushion to seal the mask to various face shapes, and create a way to attach an easily replaceable HEPA filter (high-efficiency particulate air). The revision described in this article is the fifth.

**Figure 1.**
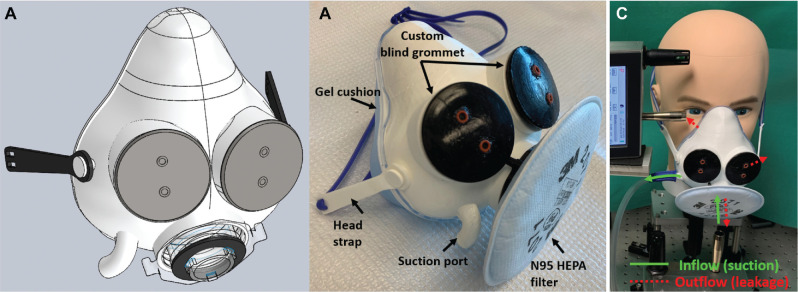
Aerosol containment mask design: (a) SolidWorks drawing, (b) final assembly of the 3-dimensional printed mask, and (c) flow directions.

**Figure 2.**
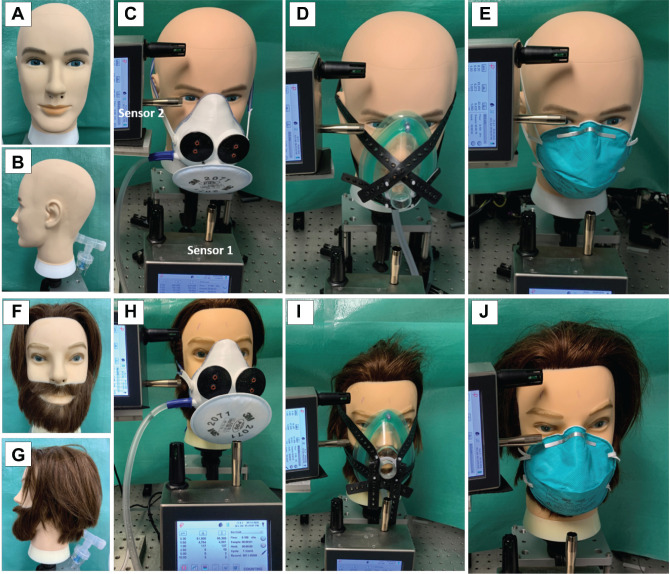
(a, b, f, g) Mannequin heads with and without hair. The (c, h) aerosol containment mask, (d, i) Ambu mask, and (e, j) a commercial N95 mask. A particle counter measured lateral (top row) and anterior (bottom row) to the mask.

The final design included a 3D printed body with 4 ports, a gel cushion for seal and comfort of fit, and custom blind grommets placed in 2 front ports plus a head strap (**Figures 1** and **2**). Each blind grommet contains 2 openings, and an endoscope or suction can be passed through any of the 4 openings. All materials can be cleaned in Cidex OPA (Advanced Sterilization Products). A N95-level commercially available respirator filter can be attached to any of the 3 front ports and replaced between patients. A suction is attached to the suction port of the mask from a commercially available suction pump.

### Testing on Model Heads vs Previously Published Designs

A test bench (**Figure 1**) was created to test the performance of the ACM in a controlled environment and to compare the mask with previously published designs. The test bench was set up in a small room with an unused biosafety cabinet. The room air was cleaned by closing the door and running the biosafety cabinet’s HEPA air filtration. Two mannequin heads, with and without facial hair, were attached to a nebulizer device (DeVilbiss) via a tube running through the back of the mannequin to the nose. The nebulizer was loaded with 2% sterile saline at a flow rate of 10 L/min.^14,15^ Aerosols were measured by an optical particle counter (Particles Plus) at approximately 2 cm anterior to the mask (sensor 1) and at 2 cm lateral to the mask near the area where its edge was against the face of the mannequin (sensor 2).

The level of aerosol around the ACM was compared with that from an Ambu mask design^12^ (an Ambu mask fitted with suction tubing leading to HEPA suction) and a commercially available N95 mask.^8,16^ The flow rate was varied for the ACM and Ambu mask designs, but the N95 mask does not have the ability to apply suction. The amount of aerosol was measured at baseline for all 3 mask designs and at various flow rates for the Ambu and ACM, between –18.5 and 16.5 L/min, by varying the valve in front of the suction pump. Particles were measured for 2 minutes with 15-second sampling intervals, and each measurement was performed 5 times for each mask type.

### Testing on Model Heads While Measuring Particle Counts Inside the Mask

**Figure 3.**
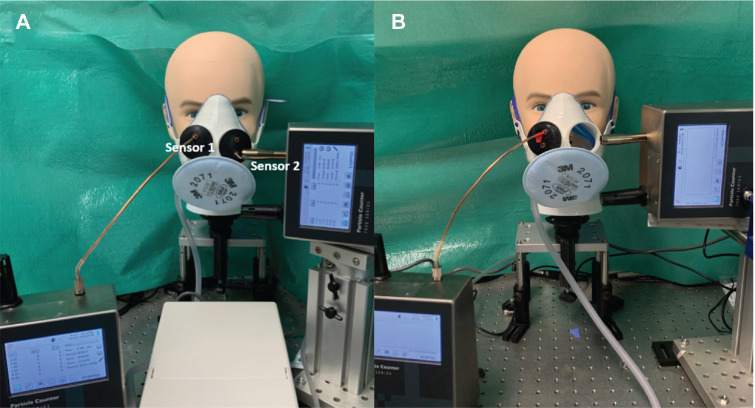
Mask testing setup for the mannequin: (a) the “closed” setup and (b) the “open” setup with the right grommet removed.

The mannequin testing was repeated for the ACM (**Figure 3a**) with a 3-mm copper tube to measure particles within the mask (sensor 1) and with a particle counter 2 cm anterior to a grommet through which a 4-mm rod was placed to mimic the placement of an endoscope (sensor 2). Measurements were made for 1 minute with a 1-second sampling interval at a flow rate of 18.5 L/min. The tests were repeated with 1 grommet uncovered, mimicking an approach that could be used to allow access for the placement of larger instruments or nasal packing.

### Statistical Analysis

Standard *t* tests, as fully specified in the text with α = 0.05, were used to test for statistical significance. All statistics were calculated with OriginLab (OriginLab Corporation, OriginPro 2021).

## Results

### Testing on Model Heads vs Previously Published Designs

**Figure 4.**
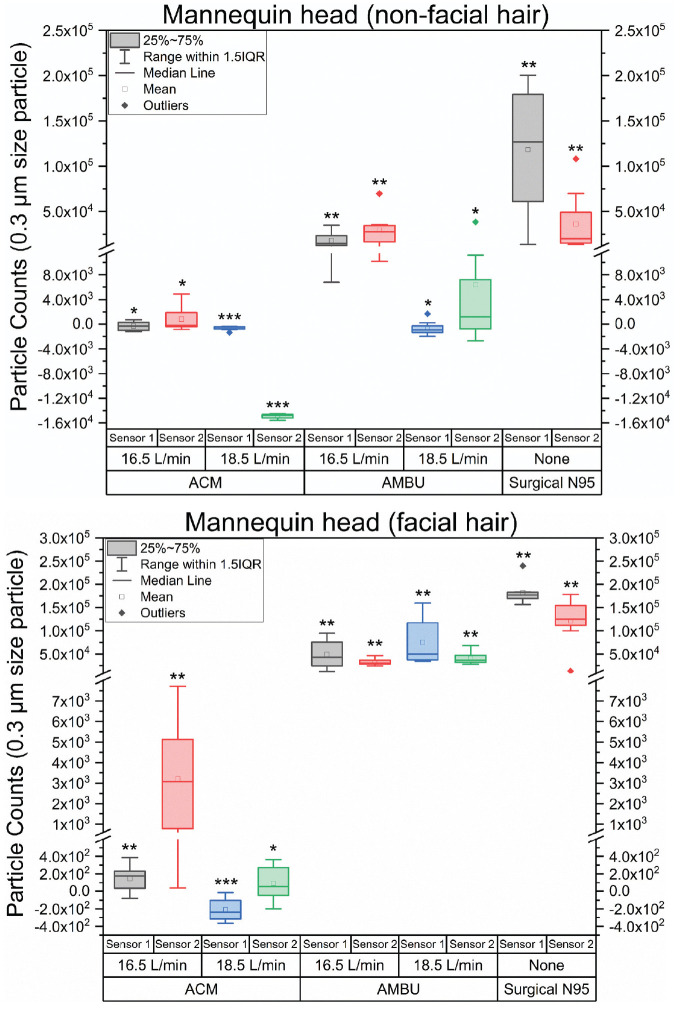
Particle counts (0.3 μm) with ambient baseline subtracted for masks on mannequins with and without facial hair. *Mean not significantly different from zero. **Positive mean significantly different from zero. ***Negative mean significantly different from zero. ACM, aerosol containment mask; IQR, interquartile range.

**Figure 5.**
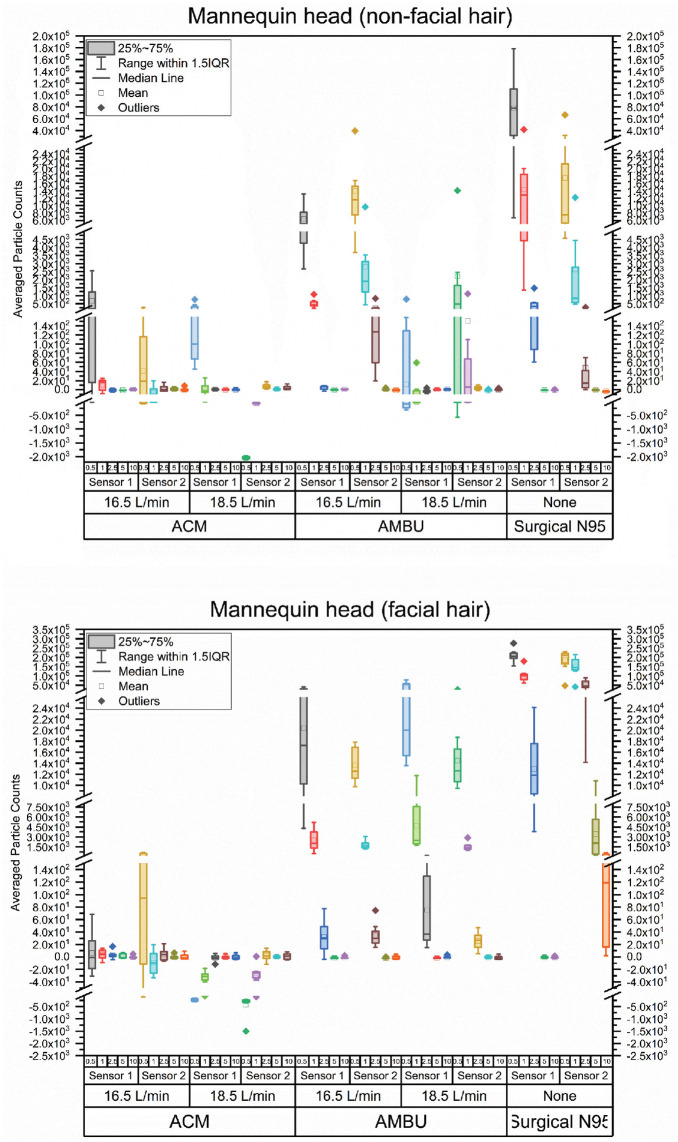
Particle counts for all particle sizes >0.3 μm with ambient baseline subtracted for the ACM, Ambu, and surgical N95 mask on mannequins with and without facial hair. ACM, aerosol containment mask; IQR, interquartile range.

**Figure 4** is a set of box plots showing average 0.3-μm particle counts (averaged over 120 seconds) for the mannequin head with and without facial hair. The average was measured 8 times. The largest changes observed were in the 0.3-μm particle; hence, only the results for this size are shown in **Figure 3** for simplicity (others are in **Figure 5**). A 2-tailed unpaired *t* test was used to test if the mean average particle count minus the baseline particle count was significantly different from zero (n = 8; Supplemental Table S1, available online). A positive mean indicates leakage of aerosols. A negative mean implies that the mask is functionally cleaning the air near the sensor. On the mannequin head with no facial hair, no leakage was found under any conditions for the ACM. At 18.5 L/min, the sensor directly in front of the N95 filter (sensor 2) had a negative mean. This implies that the air near this sensor is cleaner when the suction is on. It may be that the filter is removing particles from the air in the vicinity of the sensor or that the air flow into the mask is drawing cleaner air into the room. The Ambu mask shows leakage at both sensors when the suction is 16.5 L/min but only at sensor 2 when the suction is –18.5 L/min. The N95 shows leakage at both sensors. On the mannequin head with facial hair, the ACM shows leakage when the suction is 16.5 L/min but not when the suction is set to 18.5 L/min, while the Ambu and surgical N95 masks show leakage under all conditions.

### Testing on Model Heads While Measuring Particle Counts Inside the Mask

**Figure 6.**
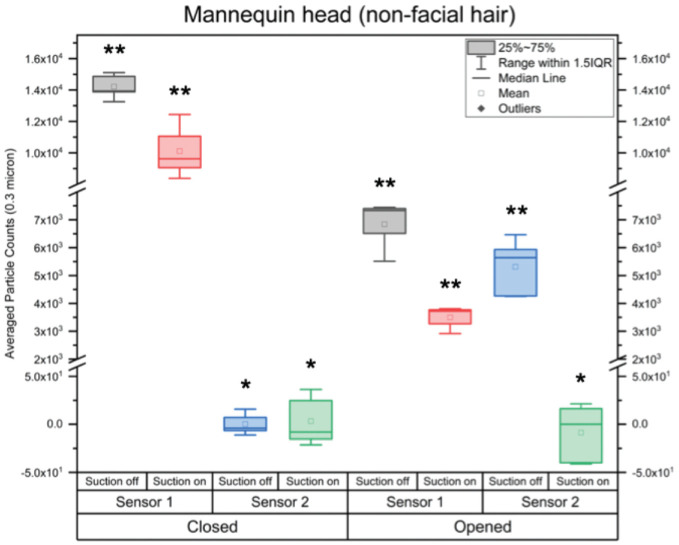
Box plots of 0.3-μm averaged particle counts. Sensor 1 (within mask). **Significant difference based on suction. Sensor 2 (outside mask). *No significant difference except with grommet removed and suction off. IQR, interquartile range.

In these experiments, the particle count from the nebulizer had to be reduced as compared with the aforementioned experiments to avoid saturating sensor 1, which was measuring the count inside the mask. Nevertheless, the particle counts exceeded any of those measured on the human volunteers. A baseline was acquired just prior to turning on the nebulizer. This was subtracted from particle counts measured with the nebulizer on, the mask suction on and off, and the grommet opened and closed. The box plots in **Figure 6** represent average particle counts measured in 5 trials for 0.3-μm particles. The data used to build the plot are in Supplemental Table S2 (available online), with box plots of the other particle sizes in Supplemental Figure S1. Sensor 1 (within mask) showed a significant difference in average particle count with the suction on and off for both experiments. Sensor 2 (outside mask) showed no significant difference with the suction on or off for the mock endoscope experiment. Hence, in this configuration, even with a very high particle count within the mask, there was no detectable leakage with the suction on or off. In the experiment with the grommet removed, there was no significant difference with the suction on. As expected, though, when the suction is turned off, there is a significant increase in particle count, as aerosols leak from the mask.

## Discussion

The ACM significantly decreased the spread of aerosol particles in mannequin testing. It outperformed previously described masks, especially on mannequins with facial hair.

Prior studies found that regular surgical masks are insufficient in protecting against aerosol escape generated by sneezing.^8^ However, an N95 respirator with an incision lined with a cut piece of surgical glove (VENT modification [valved endoscopy of the nose and throat]) contained aerosol spread.^8^ When trialed on the mannequin, the unmodified N95 underperformed when compared with the devices with suction, especially on patients with facial hair. Other barrier devices, such as a hood or box, have been detailed but may be difficult to place, are not conducive to rigid endoscopy, and do not contain suction.^17,18^

Prior studies of endoscopic surgery found that the addition of suction prevents the spread of aerosols. Dharmarajan et al found that, even in a cadaver model with an endotracheal tube in the nasopharynx attached to a nebulizer with B2 solution, no aerosols were detected visually or with a cascade impactor once a flexible suction was placed in the nasal cavity or the nasopharynx.^15^ Similarly, no aerosols were detected with drilling of a cadaveric specimen or 3D sinonasal model once a flexible suction was placed, likely because the aerosols were directed toward the suction tip rather than exiting the nares.^15^ Similarly, Workman et al did not identify aerosol contamination when utilizing the microdebrider, which is attached to suction.^8,16^ Our findings are similar in that once suction is placed on the mask at a level sufficient to overcome the generation of the aerosols and the difficulties of fit with facial hair, then no particles are detected leaking from the mask.

Creating negative pressure microenvironments around the patient to contain particles has been described. Prior studies also detailed box-like containers that can be placed around the patient for outpatient procedures or intubation, although these may be time-consuming in an outpatient setting.^11,19^ Finally, modifications of existing masks, such as the Ambu mask or nasotracheal intubation masks, have been outlined.^11,12^ While the Ambu mask outperformed an unmodified N95 mask, it was difficult to place and allowed for less access to the nasal cavity and oropharynx as compared with the ACM.

Additionally, prior studies have reported on the creation of 3D printed devices to contain aerosols. Two studies described a 3D printed vent that could be placed through a regular surgical mask, but the efficacy of these devices may be limited, as Workman et al found that surgical masks contain aerosolized particles poorly.^8,20,21^ One of these articles also detailed a complete 3D printed mask; however, it did not include suction, had only a single midline port for flexible endoscopes, and had not been tested on a human.^21^

The current study design has several limitations. The mask material is not clear. This necessitates scope guidance via a camera or the eye piece to drive the scope from the entrance of the mask into the nares. Future versions of the mask could be made with clear material through injection molding or chemical polishing of transparent 3D printed parts. While the mask allows access to the nose and oral cavity for diagnostic purposes and single-instrument procedures, such as suctioning and hand instruments, it does not allow for insertion of larger objects (eg, nasal packing) without removing 1 of the grommets. Nevertheless, in a trial on the mannequin with the grommet off, we found no significant increase in aerosols external to the mask with the suction on (**Figure 4**). It may be possible to uncover a grommet to get wide exposure while providing good protection to the health care worker. Additionally, in the Workman et al study of the N95 mask with VENT modification, some contamination occurred after N95 respirator removal.^8^ We have not yet tested removal procedures, although we believe that most aerosols would be evacuated by the suction pump.

Studies are ongoing regarding whether a single suction pump, such as that from an SMR cart (Global Surgical Corporation), can be split and continue to provide adequate suction to the mask and a surgical suction. This article describes testing of the mask on only a mannequin, but we have tested it on healthy volunteers and are in the process of completing a larger-scale clinical trial on patients presenting to an otolaryngology clinic.^22^

## Conclusion

A negative pressure mask may allow for the passage of rigid and flexible endoscopes without leakage of particles outside the mask. This may help prevent contamination of the room and protect health care workers during viral pandemics that involve airborne contagion. A larger clinical study is ongoing.

## Supplemental Material

sj-docx-2-oto-10.1177_01945998211024944 – Supplemental material for COVID-19 in the Clinic: Aerosol Containment Mask for Endoscopic Otolaryngologic Clinic Procedures Click here for additional data file. Supplemental material, sj-docx-2-oto-10.1177_01945998211024944 for COVID-19 in the Clinic: Aerosol Containment Mask for Endoscopic Otolaryngologic Clinic Procedures by Elisabeth H. Ference, Wihan Kim, John S. Oghalai, Jee-Hong Kim and Brian E. Applegate in Otolaryngology–Head and Neck Surgery

sj-pdf-1-oto-10.1177_01945998211024944 – Supplemental material for COVID-19 in the Clinic: Aerosol Containment Mask for Endoscopic Otolaryngologic Clinic Procedures Click here for additional data file. Supplemental material, sj-pdf-1-oto-10.1177_01945998211024944 for COVID-19 in the Clinic: Aerosol Containment Mask for Endoscopic Otolaryngologic Clinic Procedures by Elisabeth H. Ference, Wihan Kim, John S. Oghalai, Jee-Hong Kim and Brian E. Applegate in Otolaryngology–Head and Neck Surgery
