# Supplementary material for: COVID-19 in the Clinic: Aerosol Containment Mask for Endoscopic Otolaryngologic Clinic Procedures
Source: Otolaryngol Head Neck Surg. 2021 Jun 22;166(5):850–7. doi: 10.1177/01945998211024944 (PMC8262032; doi:10.1177/01945998211024944)
Supplement: sj-docx-2-oto-10.1177_01945998211024944 – Supplemental material for COVID-19 in the Clinic: Aerosol Containment Mask for Endoscopic Otolaryngologic Clinic Procedures [file sj-docx-2-oto-10.1177_01945998211024944.docx]

Supplemental Table 1

| Condition | Mask | Flow rate | Location | Size | Mean | SD | SEM | P value | Label |
| --- | --- | --- | --- | --- | --- | --- | --- | --- | --- |
| Non-facial hair | ACM | 16.5 L/min | Sensor 1 | 0.3 µm | -327.25 | 746.9234 | 264.0773 | 0.2552 | * |
|  |  |  |  | 0.5 µm | 674.125 | 950.6906 | 336.1199 | 0.0849 | * |
|  |  |  |  | 1 µm | 11.125 | 12.48714 | 4.41487 | 0.0398 | ** |
|  |  |  |  | 2.5 µm | -0.875 | 2.44949 | 0.86603 | 0.3460 | * |
|  |  |  |  | 5 µm | -1 | 0.74402 | 0.26305 | 0.0067 | *** |
|  |  |  |  | 10 µm | 0.625 | 1.41421 | 0.5 | 0.2515 | * |
|  |  |  | Sensor 2 | 0.3 µm | 817.375 | 1966.976 | 695.4309 | 0.2783 | * |
|  |  |  |  | 0.5 µm | 40.75 | 125.4703 | 44.36044 | 0.3889 | * |
|  |  |  |  | 1 µm | -6.125 | 14.12634 | 4.99442 | 0.2597 | * |
|  |  |  |  | 2.5 µm | 2.5 | 7.1101 | 2.5138 | 0.3531 | * |
|  |  |  |  | 5 µm | 1.5 | 2.81577 | 0.99553 | 0.1756 | * |
|  |  |  |  | 10 µm | 0.125 | 3.90741 | 1.38148 | 0.9304 | * |
|  |  | 18.5 L/min | Sensor 1 | 0.3 µm | -644.5 | 336.6058 | 119.0081 | 0.0010 | *** |
|  |  |  |  | 0.5 µm | 222.25 | 255.422 | 90.30532 | 0.0434 | ** |
|  |  |  |  | 1 µm | 2 | 12.18899 | 4.30946 | 0.6567 | * |
|  |  |  |  | 2.5 µm | 0.875 | 1.75255 | 0.61962 | 0.2008 | * |
|  |  |  |  | 5 µm | 0.375 | 1.12599 | 0.3981 | 0.3776 | * |
|  |  |  |  | 10 µm | 0.375 | 1.76777 | 0.625 | 0.5674 | * |
|  |  |  | Sensor 2 | 0.3 µm | -14912.9 | 393.5036 | 139.1245 | 0.0000 | *** |
|  |  |  |  | 0.5 µm | -2043 | 54.93616 | 19.42287 | 0.0000 | *** |
|  |  |  |  | 1 µm | -62.25 | 18.19537 | 6.43303 | 0.0000 | *** |
|  |  |  |  | 2.5 µm | 7.5 | 5.25765 | 1.85886 | 0.0050 | ** |
|  |  |  |  | 5 µm | 1.75 | 1.99553 | 0.70553 | 0.0422 | ** |
|  |  |  |  | 10 µm | 4 | 4.47214 | 1.58114 | 0.0392 | ** |
|  |  |  |  |  |  |  |  |  |  |
|  | AMBU | 16.5 L/min | Sensor 1 | 0.3 µm | 17721.38 | 9061.714 | 3203.8 | 0.0009 | ** |
|  |  |  |  | 0.5 µm | 6348.375 | 3370.88 | 1191.786 | 0.0011 | ** |
|  |  |  |  | 1 µm | 516 | 267.2869 | 94.50019 | 0.0009 | ** |
|  |  |  |  | 2.5 µm | 4.75 | 4.30116 | 1.52069 | 0.0168 | ** |
|  |  |  |  | 5 µm | 1.39E-17 | 1.0351 | 0.36596 | 1.0000 | * |
|  |  |  |  | 10 µm | 0.75 | 1.06904 | 0.37796 | 0.0876 | * |
|  |  |  | Sensor 2 | 0.3 µm | 29494.38 | 18610.93 | 6579.956 | 0.0029 | ** |
|  |  |  |  | 0.5 µm | 13941.25 | 11127.81 | 3934.274 | 0.0094 | ** |
|  |  |  |  | 1 µm | 2814.375 | 2907.562 | 1027.978 | 0.0290 | ** |
|  |  |  |  | 2.5 µm | 211 | 264.3885 | 93.47544 | 0.0586 | * |
|  |  |  |  | 5 µm | 0.625 | 1.75255 | 0.61962 | 0.3467 | * |
|  |  |  |  | 10 µm | -0.875 | 1.59799 | 0.56497 | 0.1654 | * |
|  |  | 18.5 L/min | Sensor 1 | 0.3 µm | -682.75 | 1149.462 | 406.3961 | 0.1368 | * |
|  |  |  |  | 0.5 µm | 11.875 | 355.0434 | 125.5268 | 0.9273 | * |
|  |  |  |  | 1 µm | -16 | 36.74599 | 12.99167 | 0.2579 | * |
|  |  |  |  | 2.5 µm | -3.125 | 3.54562 | 1.25357 | 0.0414 | *** |
|  |  |  |  | 5 µm | 0.5 | 1.38873 | 0.49099 | 0.3424 | * |
|  |  |  |  | 10 µm | 0.75 | 1.12599 | 0.3981 | 0.1016 | * |
|  |  |  | Sensor 2 | 0.3 µm | 6362.196 | 13615.22 | 4813.709 | 0.2278 | * |
|  |  |  |  | 0.5 µm | 2202.964 | 4861.789 | 1718.902 | 0.2408 | * |
|  |  |  |  | 1 µm | 150.4464 | 396.6331 | 140.231 | 0.3189 | * |
|  |  |  |  | 2.5 µm | 3.94643 | 4.06861 | 1.43847 | 0.0288 | ** |
|  |  |  |  | 5 µm | -1.58929 | 1.3562 | 0.47949 | 0.0129 | *** |
|  |  |  |  | 10 µm | 0.42857 | 1.51186 | 0.53452 | 0.4490 | * |
|  |  |  |  |  |  |  |  |  |  |
|  | Surgical N95 | None | Sensor 1 | 0.3 µm | 118508.6 | 69914.56 | 24718.53 | 0.0020 | ** |
|  |  |  |  | 0.5 µm | 78076.5 | 56557.71 | 19996.17 | 0.0059 | ** |
|  |  |  |  | 1 µm | 14294.88 | 12935.46 | 4573.375 | 0.0167 | ** |
|  |  |  |  | 2.5 µm | 435.375 | 465.5037 | 164.5804 | 0.0332 | ** |
|  |  |  |  | 5 µm | -0.875 | 0.75593 | 0.26726 | 0.0136 | *** |
|  |  |  |  | 10 µm | -0.875 | 0.74402 | 0.26305 | 0.0127 | *** |
|  |  |  | Sensor 2 | 0.3 µm | 36111 | 34507.89 | 12200.38 | 0.0021 | ** |
|  |  |  |  | 0.5 µm | 17371.25 | 21707.81 | 7674.869 | 0.0058 | ** |
|  |  |  |  | 1 µm | 2625.375 | 4058.522 | 1434.904 | 0.0011 | ** |
|  |  |  |  | 2.5 µm | 48.625 | 90.59476 | 32.03008 | 0.0017 | ** |
|  |  |  |  | 5 µm | -0.5 | 1.45774 | 0.51539 | 0.0036 | ** |
|  |  |  |  | 10 µm | -3.5 | 1.16496 | 0.41188 | 0.0001 | ** |
|  |  |  |  |  |  |  |  |  |  |
| Facial hair | ACM | 16.5 L/min | Sensor 1 | 0.3 µm | 148 | 147.1132 | 52.01236 | 0.0249 | ** |
|  |  |  |  | 0.5 µm | 6.375 | 33.01082 | 11.67109 | 0.6019 | * |
|  |  |  |  | 1 µm | 4 | 8.37407 | 2.96068 | 0.2187 | * |
|  |  |  |  | 2.5 µm | 3.375 | 6.09303 | 2.15421 | 0.1612 | * |
|  |  |  |  | 5 µm | 1.125 | 3.77018 | 1.33296 | 0.4266 | * |
|  |  |  |  | 10 µm | -0.125 | 2.10017 | 0.74252 | 0.8711 | * |
|  |  |  | Sensor 2 | 0.3 µm | 3215.75 | 2807.977 | 992.7699 | 0.0143 | ** |
|  |  |  |  | 0.5 µm | 223.375 | 306.0307 | 108.1982 | 0.0779 | * |
|  |  |  |  | 1 µm | -9.25 | 19.36123 | 6.84523 | 0.2186 | * |
|  |  |  |  | 2.5 µm | 3.75 | 9.41029 | 3.32704 | 0.2968 | * |
|  |  |  |  | 5 µm | -0.25 | 3.02372 | 1.06904 | 0.8218 | * |
|  |  |  |  | 10 µm | 0.375 | 4.23421 | 1.49702 | 0.8094 | * |
|  |  | 18.5 L/min | Sensor 1 | 0.3 µm | -211.625 | 130.7117 | 46.21357 | 0.0026 | *** |
|  |  |  |  | 0.5 µm | -232 | 51.32651 | 18.14666 | 0.0000 | *** |
|  |  |  |  | 1 µm | -34.625 | 15.0327 | 5.31486 | 0.0003 | *** |
|  |  |  |  | 2.5 µm | -1.375 | 5.01248 | 1.77218 | 0.4632 | * |
|  |  |  |  | 5 µm | -0.125 | 2.6152 | 0.92461 | 0.8963 | * |
|  |  |  |  | 10 µm | -0.375 | 3.70328 | 1.30931 | 0.7829 | * |
|  |  |  | Sensor 2 | 0.3 µm | -2420.75 | 7225.451 | 2554.583 | 0.3749 | * |
|  |  |  |  | 0.5 µm | -422.625 | 438.2484 | 154.9442 | 0.0294 | *** |
|  |  |  |  | 1 µm | -34.375 | 32.0925 | 11.34641 | 0.0191 | *** |
|  |  |  |  | 2.5 µm | 2.25 | 8.2017 | 2.89974 | 0.4632 | * |
|  |  |  |  | 5 µm | 0.5 | 1.84681 | 0.65295 | 0.4689 | * |
|  |  |  |  | 10 µm | 1 | 4.78091 | 1.69031 | 0.5727 | * |
|  |  |  |  |  |  |  |  |  |  |
|  | AMBU | 16.5 L/min | Sensor 1 | 0.3 µm | 49198.63 | 30964.52 | 10947.61 | 0.0028 | ** |
|  |  |  |  | 0.5 µm | 20377.25 | 13570.08 | 4797.746 | 0.0038 | ** |
|  |  |  |  | 1 µm | 2496.875 | 1659.743 | 586.8078 | 0.0038 | ** |
|  |  |  |  | 2.5 µm | 32.25 | 27.53699 | 9.7358 | 0.0129 | ** |
|  |  |  |  | 5 µm | -1.25 | 1.38873 | 0.49099 | 0.0383 | *** |
|  |  |  |  | 10 µm | -1 | 1.48805 | 0.5261 | 0.0991 | * |
|  |  |  | Sensor 2 | 0.3 µm | 32695.13 | 7428.298 | 2626.3 | 0.0000 | ** |
|  |  |  |  | 0.5 µm | 13637.63 | 3164.751 | 1118.908 | 0.0000 | ** |
|  |  |  |  | 1 µm | 1812.75 | 611.8599 | 216.3252 | 0.0001 | ** |
|  |  |  |  | 2.5 µm | 34.75 | 18.96943 | 6.70671 | 0.0013 | ** |
|  |  |  |  | 5 µm | -1.75 | 0.92582 | 0.32733 | 0.0011 | ** |
|  |  |  |  | 10 µm | -0.875 | 3.11391 | 1.10093 | 0.4528 | * |
|  |  | 18.5 L/min | Sensor 1 | 0.3 µm | 75271.25 | 50581.77 | 17883.36 | 0.0040 | ** |
|  |  |  |  | 0.5 µm | 34890 | 27714.99 | 9798.73 | 0.0092 | ** |
|  |  |  |  | 1 µm | 4692.375 | 3992.874 | 1411.694 | 0.0127 | ** |
|  |  |  |  | 2.5 µm | 75.375 | 73.45929 | 25.97178 | 0.0229 | ** |
|  |  |  |  | 5 µm | -1.5 | 1.12599 | 0.3981 | 0.0070 | ** |
|  |  |  |  | 10 µm | -0.375 | 1.72689 | 0.61055 | 0.5585 | * |
|  |  |  | Sensor 2 | 0.3 µm | 40858.13 | 13348.66 | 4719.466 | 0.0001 | ** |
|  |  |  |  | 0.5 µm | 14486.13 | 5733.946 | 2027.256 | 0.0002 | ** |
|  |  |  |  | 1 µm | 1538.75 | 599.6087 | 211.9937 | 0.0002 | ** |
|  |  |  |  | 2.5 µm | 25.75 | 13.73993 | 4.8578 | 0.0011 | ** |
|  |  |  |  | 5 µm | -0.5 | 1.51186 | 0.53452 | 0.3807 | * |
|  |  |  |  | 10 µm | -1.125 | 2.76457 | 0.97742 | 0.2875 | * |
|  |  |  |  |  |  |  |  |  |  |
|  | Surgical N95 | None | Sensor 1 | 0.3 µm | 181807.4 | 25036.59 | 8851.77 | 0.0000 | ** |
|  |  |  |  | 0.5 µm | 209409.1 | 34675.41 | 12259.61 | 0.0000 | ** |
|  |  |  |  | 1 µm | 103302.3 | 35140.09 | 12423.9 | 0.0001 | ** |
|  |  |  |  | 2.5 µm | 12962.25 | 7151.556 | 2528.457 | 0.0014 | ** |
|  |  |  |  | 5 µm | -0.625 | 1.66905 | 0.5901 | 0.3247 | * |
|  |  |  |  | 10 µm | -0.75 | 1.18773 | 0.41993 | 0.1173 | * |
|  |  |  | Sensor 2 | 0.3 µm | 121663.4 | 50480.87 | 17847.68 | 0.0002 | ** |
|  |  |  |  | 0.5 µm | 184375.5 | 61104.49 | 21603.7 | 0.0001 | ** |
|  |  |  |  | 1 µm | 148808.9 | 54428.67 | 19243.44 | 0.0001 | ** |
|  |  |  |  | 2.5 µm | 52311.5 | 25764.86 | 9109.253 | 0.0007 | ** |
|  |  |  |  | 5 µm | 3434.125 | 4047.888 | 1431.145 | 0.0475 | ** |
|  |  |  |  | 10 µm | 210.375 | 236.3948 | 83.57818 | 0.0400 | ** |

Supplemental Table 2:

| Condition | Location | Suction | Size | Mean | SD | SEM | P-value | Label |
| --- | --- | --- | --- | --- | --- | --- | --- | --- |
| Closed | Sensor 1 | Off | 0.3 µm | 14360.34 | 774.1399 | 346.2059 | 0.0000 | ** |
|  |  |  | 0.5 µm | 14110.49 | 1104.845 | 494.1016 | 0.0000 | ** |
|  |  |  | 1 µm | 6684.727 | 1036.931 | 463.7295 | 0.0001 | ** |
|  |  |  | 2.5 µm | 981.5933 | 180.3811 | 80.6689 | 0.0003 | ** |
|  |  |  | 5 µm | 6.90667 | 1.25988 | 0.56344 | 0.0002 | ** |
|  |  |  | 10 µm | 0.25 | 0.20983 | 0.09384 | 0.1616 | * |
|  |  | On | 0.3 µm | 10261.24 | 1621.578 | 725.1917 | 0.0002 | ** |
|  |  |  | 0.5 µm | 5506.343 | 1111.015 | 496.861 | 0.0004 | ** |
|  |  |  | 1 µm | 1803.367 | 352.0125 | 157.4248 | 0.0003 | ** |
|  |  |  | 2.5 µm | 309.2033 | 66.51611 | 29.74691 | 0.0005 | ** |
|  |  |  | 5 µm | 2.83333 | 0.3864 | 0.1728 | 0.0000 | ** |
|  |  |  | 10 µm | 0.09667 | 0.06604 | 0.02953 | 0.3965 | * |
|  | Sensor 2 | Off | 0.3 µm | 253.3467 | 20.79125 | 9.29813 | 0.9670 | * |
|  |  |  | 0.5 µm | 20.72667 | 1.73272 | 0.77489 | 0.4649 | * |
|  |  |  | 1 µm | 2.45667 | 0.41072 | 0.18368 | 0.3167 | * |
|  |  |  | 2.5 µm | 0.34667 | 0.04625 | 0.02068 | 0.5821 | * |
|  |  |  | 5 µm | 0.01667 | 0.01179 | 0.00527 | 0.0978 | * |
|  |  |  | 10 µm | 0.01491 | 0.00667 | 0.05 | 0.7725 | * |
|  |  | On | 0.3 µm | 256.3867 | 23.64117 | 10.57265 | 0.7907 | * |
|  |  |  | 0.5 µm | 21.25667 | 2.02085 | 0.90375 | 0.1824 | * |
|  |  |  | 1 µm | 2.82 | 0.74707 | 0.3341 | 0.0546 | * |
|  |  |  | 2.5 µm | 0.38333 | 0.0874 | 0.03909 | 1.0000 | * |
|  |  |  | 5 µm | 0.04333 | 0.04183 | 0.01871 | 0.2302 | * |
|  |  |  | 10 µm | 0.05667 | 0.03837 | 0.01716 | 0.8558 | * |
|  |  |  |  |  |  |  |  |  |
| Opened | Sensor 1 | Off | 0.3 µm | 7493.187 | 763.9493 | 341.6485 | 0.0001 | ** |
|  |  |  | 0.5 µm | 3130.09 | 380.5897 | 170.2049 | 0.0001 | ** |
|  |  |  | 1 µm | 744.9833 | 79.42194 | 35.51857 | 0.0000 | ** |
|  |  |  | 2.5 µm | 744.9833 | 79.42194 | 35.51857 | 0.0000 | ** |
|  |  |  | 5 µm | 0.6 | 0.35649 | 0.15943 | 0.0304 | ** |
|  |  |  | 10 µm | 0.11667 | 0.10541 | 0.04714 | 0.2509 | * |
|  |  | On | 0.3 µm | 4149.77 | 329.5099 | 147.3613 | 0.0000 | ** |
|  |  |  | 0.5 µm | 1500.723 | 151.2504 | 67.64122 | 0.0000 | ** |
|  |  |  | 1 µm | 345.0967 | 28.48167 | 12.73739 | 0.0000 | ** |
|  |  |  | 2.5 µm | 38.50333 | 4.57967 | 2.04809 | 0.0000 | ** |
|  |  |  | 5 µm | 0.64333 | 0.77043 | 0.34454 | 0.1656 | * |
|  |  |  | 10 µm | 0.25 | 0.42048 | 0.18805 | 0.3131 | * |
|  | Sensor 2 | Off | 0.3 µm | 5947.96 | 926.7702 | 414.4642 | 0.0003 | ** |
|  |  |  | 0.5 µm | 4815.023 | 2032.256 | 908.8525 | 0.0064 | ** |
|  |  |  | 1 µm | 2177.973 | 1295.104 | 579.1879 | 0.0199 | ** |
|  |  |  | 2.5 µm | 641.3733 | 509.1112 | 227.6815 | 0.0481 | ** |
|  |  |  | 5 µm | 78.74667 | 74.17287 | 33.17112 | 0.0767 | * |
|  |  |  | 10 µm | 0.27849 | 0.12454 | 0.53333 | 0.0578 | * |
|  |  | On | 0.3 µm | 627.3867 | 81.83634 | 36.59832 | 0.5555 | * |
|  |  |  | 0.5 µm | 159.29 | 209.7728 | 93.81326 | 0.3485 | * |
|  |  |  | 1 µm | 18.05667 | 27.4233 | 12.26407 | 0.3050 | * |
|  |  |  | 2.5 µm | 2.18 | 3.10518 | 1.38868 | 0.3136 | * |
|  |  |  | 5 µm | 0.22667 | 0.40579 | 0.18148 | 0.0765 | * |
|  |  |  | 10 µm | 0.02 | 0.02173 | 0.00972 | 0.0547 | * |
