# Supplementary figures and images for: COVID-19 in the Clinic: Aerosol Containment Mask for Endoscopic Otolaryngologic Clinic Procedures
Source: Otolaryngol Head Neck Surg. 2021 Jun 22;166(5):850–7. doi: 10.1177/01945998211024944 (PMC8262032; doi:10.1177/01945998211024944)

# Mannequin head (non-facial hair)

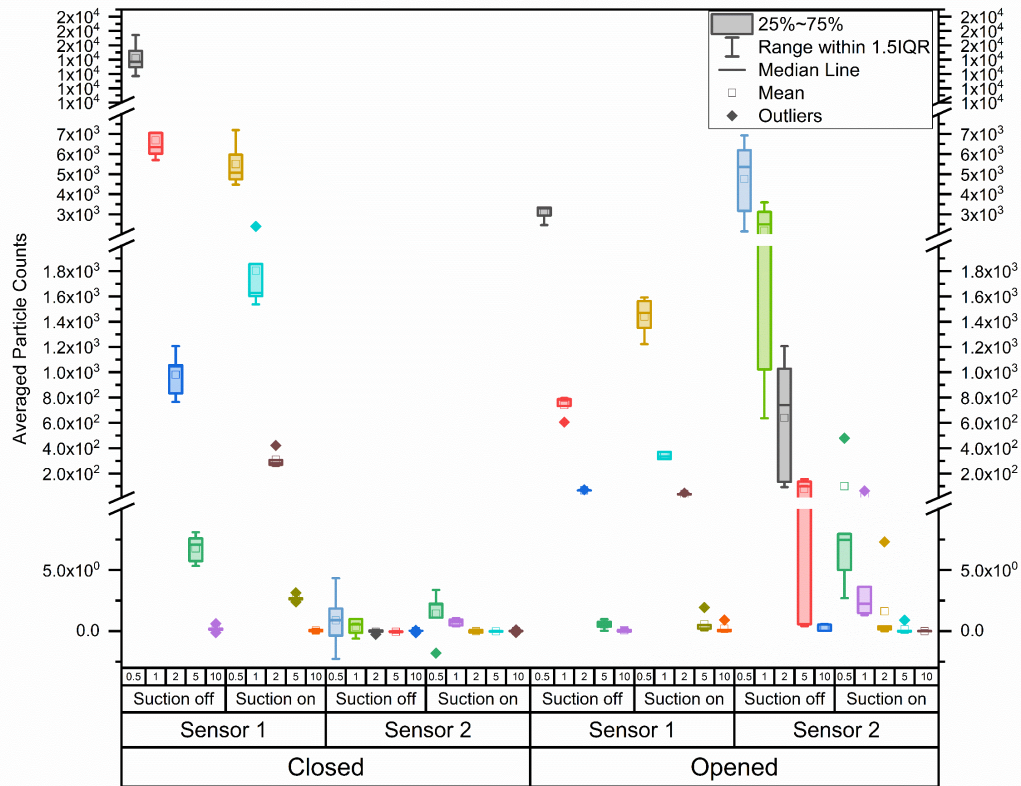

Supplement: sj-pdf-1-oto-10.1177_01945998211024944 – Supplemental material for COVID-19 in the Clinic: Aerosol Containment Mask for Endoscopic Otolaryngologic Clinic Procedures [file sj-pdf-1-oto-10.1177_01945998211024944.pdf]
